# Influence of Socio-Economic and Psychosocial Profiles on the Human Breast Milk Bacteriome of South African Women

**DOI:** 10.3390/nu11061390

**Published:** 2019-06-20

**Authors:** Anna Ojo-Okunola, Shantelle Claassen-Weitz, Kilaza S. Mwaikono, Sugnet Gardner-Lubbe, Dan J. Stein, Heather J. Zar, Mark P. Nicol, Elloise du Toit

**Affiliations:** 1 Division of Medical Microbiology, Department of Pathology, Faculty of Health Sciences, Observatory 7925, University of Cape Town, Cape Town 7700, South Africa; tellafiela@gmail.com (S.C.-W.); mark.nicol@uwa.edu.au (M.P.N.); elloisedutoit@gmail.com (E.d.T.); 2 Computational Biology Group and H3ABioNet, Department of Integrative Biomedical Sciences, Observatory 7925, University of Cape Town, Cape Town 7700, South Africa; kilazasmsn24@gmail.com; 3 Department of Science and Laboratory Technology, Dar es Salaam Institute of Technology, P. O. Box 2958, Dar es Salaam 11000, Tanzania; 4 Department of Statistics and Actuarial Science, Faculty of Economic and Management Sciences, Stellenbosch University, Matieland 7602, Stellenbosch, South Africa; slubbe@sun.ac.za; 5 SA MRC Unit on Risk & Resilience in Mental Disorders, Dept of Psychiatry & Neuroscience Institute, Observatory 7925, University of Cape Town, Cape Town 7700, South Africa; dan.stein@uct.ac.za; 6 Institute of Infectious Disease and Molecular Medicine, Faculty of Health Sciences, Observatory 7925, University of Cape Town, Cape Town 7700, South Africa; heather.zar@uct.ac.za; 7 SAMRC Unit on Child & Adolescent Health, Observatory 7925, University of Cape Town, Cape Town 7700, South Africa; 8 Department of Pediatrics and Child Health, Red Cross War Memorial Children’s Hospital, Rondebosch, Cape Town 7700, South Africa; 9 School of Biomedical Sciences, Division of Infection and Immunity, The University of Western Australia, M504, Perth, WA 6009, Australia

**Keywords:** human breast milk, bacteriome, microbiome, 16S rRNA gene sequencing, bacterial profiles, socio-economic, psychosocial, Africa

## Abstract

The human breast milk (HBM) bacteriome is an important, continuous source of microbes to the neonate in early life, playing an important role in shaping the infant’s intestinal bacteriome. Study of the composition of the HBM bacteriome is an emerging area of research, with little information available, particularly from low- and middle-income countries. The aim of this study was to characterize the diversity of bacterial communities in HBM samples collected between 6–10 weeks postpartum from lactating South African women and to study potential influencing factors of the bacteriome. Using 16S rRNA gene sequencing of samples from 554 women, we demonstrated that the HBM bacteriome was largely dominated by the phyla Firmicutes (mean relative abundance: 71.1%) and Actinobacteria (mean relative abundance: 16.4%). The most abundant genera identified from the HBM bacteriome were *Streptococcus* (mean relative abundance: 48.6%), *Staphylococcus* (mean relative abundance: 17.8%), *Rothia* (mean relative abundance: 5.8%), and *Corynebacterium* (mean relative abundance: 4.3%). “Core” bacterial genera including *Corynebacterium*, *Streptococcus, Staphylococcus, Rothia, Veillonella, Gemella, Acinetobacter, Micrococcus* and a genus belonging to the Enterobacteriaceae family were present in 80% of samples. HBM samples were classified, according to their bacteriome, into three major clusters, dominated by the genera *Staphylococcus* (cluster 1), a combination of *Staphylococcus* and *Streptococcus* (cluster 2), and *Streptococcus* (cluster 3). The cluster groups differed significantly for Shannon and chao1 richness indices. Bacterial interactions were studied using co-occurrence networks with positive associations observed between the abundances of *Staphylococcus* and *Corynebacteria* (members of the skin microflora) and between *Streptococcus, Rothia, Veillonella*, and *Gemella* (members of the oral microflora). HBM from older mothers had a higher Shannon diversity index. The study site was associated with differences in HBM bacteriome composition (permutational multivariate analysis of variance using distance matrices (PERMANOVA), *p* < 0.05). No other tested socio-demographic or psychosocial factors were associated with HBM bacterial composition.

## 1. Introduction

The bacterial community (the bacteriome) present in human breast milk (HBM) is diverse and plays an important role in the health of both mothers and their infants [1,2]. HBM bacteria are one of the main sources of continuous microbes to the infant in early life and play a role in shaping the infant’s intestinal bacteriome [3]. Several bacterial genera, including *Streptococcus, Staphylococcus, Corynebacterium, Pseudomonas,* and lactic acid bacteria (LAB; *Lactobacillus* spp. and *Bifidobacterium* spp.) have been identified as key components of the HBM bacteriome, with the phyla Firmicutes and Proteobacteria typically dominating [4,5,6].

The concept of a core bacteriome has been proposed [7], with *Staphylococcus* and *Streptococcus* uniformly present [2,8,9], but with variation in other genera [10]. Despite a core bacteriome, substantial variability in HBM bacterial profiles occurs and has been linked to various exposures. For example, HBM from women who delivered vaginally showed a different bacterial profile compared to those who had caesarean section delivery [6]. Other factors, such as geographical location, maternal weight and body mass index, maternal health, maternal dietary intake, and lactational stage, have also been shown to influence the HBM bacteriome composition [5,6,11,12,13]. However, previous studies have been limited by sample size (*n* = 10 to 133), with few studies having been performed in sub-Saharan Africa [5,14], and studies have not yet evaluated socio-demographic and psychosocial factors in detail.

We therefore conducted a large cross-sectional study, nested within an existing birth cohort, to describe the bacteriome of HBM from a cohort of South African women and the associated factors.

## 2. Materials and Methods

### 2.1. Study Settings: Drakenstein Child Health Study

This was a cross-sectional study nested within an existing birth cohort study, the “Drakenstein Child Health Study” (DCHS) [15]. The primary aim of the DCHS is to investigate the early life determinants of child health in two poor communities in South Africa, a low- and middle-income country (LMIC). The birth cohort is based in the Drakenstein sub-district, a semi-rural area 60 km outside Cape Town, South Africa and has an estimated population of 200,000. The study sites include the TC Newman community health clinic (which serves a population with mixed ancestry), and Mbekweni (which serves a population with predominately black African isiXhosa ancestry). These two study sites are approximately 5 km apart. Inhabitants live in informal housing or crowded conditions, with high levels of unemployment, a high prevalence of tobacco smoke exposure, alcohol misuse, malnutrition, and other poverty-related exposures [15].

### 2.2. Clinical Data and Sample Collection

Pregnant women, 18 years of age or older (*n* = 1137), were enrolled between 20 and 28 weeks of gestation and were followed up until their infants were 5 years old. Written informed consent was obtained from all participating mothers. On recruitment, detailed demographic and risk factor data were collected. Mothers underwent physical examination, and metrics such as maternal height and weight were collected [15].

HBM samples were collected from 554 women at 6–10 weeks postpartum. All participating women were asked to wash their hands, nipples, and surrounding breast area with soap and water to minimize the presence of skin bacteria. HBM was collected manually by hand expression into a sterile collection container after discarding the first few drops. After collection, the samples were transported on ice to the research laboratory at the University of Cape Town and stored at −80 °C until further processing.

Ethical approval for the present study and the parent study (DCHS) was obtained from the University of Cape Town (UCT) Human Research Ethics committee (reference numbers 649/2016 and 401/2009).

### 2.3. Bacterial Nucleic Acid Extraction and Quantification

HBM samples were homogenized by vortexing, and skim milk preparation was adapted from a previously published protocol [16]. In brief, the samples were centrifuged at 3500 g for 20 min at −10 °C, and the fat layer discarded. The supernatant was then centrifuged at 7600 g for 10 min at room temperature, and the pellet was used for DNA extraction. Total genomic DNA was extracted using the commercial manual extraction kit, ZR Fungal/Bacterial DNA MiniPrep™ (Zymo Research Corp., Irvine, CA, USA), which incorporates a bead-beading step. All bead-beating steps were performed in a TissueLyser LT (Qiagen) at a frequency of 50 Hz for 5 min.

The quantity and purity of DNA was measured using a NanoDrop™ ND-2000c Spectrophotometer (Thermo Scientific, Inc.). DNA was stored at −20 °C until further processing. The bacterial 16S rRNA gene was quantified using a 16S qPCR protocol previously described [17]. Each 30 µL PCR reaction contained 2.5 µL DNA template, 1 µL of probe (16S-P1 (FAM- ATT AGA TAC CCT GGT AGT CCA –MGB), 15 µL Sensifast Probe No-rox (BIO-86020), 9.5 µL of MilliQ water, and 1 µL each of 0.333 µM forward and reverse primer (16S-F1: 5′-CGA AAG CGT GGG GAG CAA A -3′ and 16S-R1: 5′-GTT CGT ACT CCC CAG GCG G-3′, respectively) [17]. PCR cycling conditions were as follows: 50 °C for 2 min; 95 °C for 10 min; 45 cycles of 95 °C for 15 s, 60 °C for 60 s, and 72 °C for 1 s [17].

### 2.4. Extraction and Sequencing Controls

Extraction controls were processed alongside HBM specimens and were included for each batch of extractions (Supplementary Figure S1). Positive extraction controls consisted of 1000 µL elution buffer (provided by the ZR Fungal/Bacterial DNA MiniPrep™ kit) spiked with 75 µL Zymobiomics™ microbial community standard (Catalogue no. D6300, Zymo Research Corp., Irvine, CA, USA). Negative extraction controls (NTC; no template control) consisted of 1000 µL of unspiked elution buffer. DNA extracts from negative extraction controls served as negative PCR and sequencing controls. A subset of DNA extracts from negative extraction controls were spiked with *Mycobacterium smegmatis* (*M. smegmatis*) DNA (Supplementary Figure S1B) at a 16S rRNA gene concentration similar to that obtained from HBM samples assessed by qPCR (herein referred to as “*M. smegmatis*-spiked-NTC”). *M. smegmatis*-spiked-NTC were used to correct for potential contamination, resulting from bacterial DNA in buffers and reagents, during bioinformatics processing of sequenced reads. To determine experimental reproducibility, library preparation and sequencing were carried out in duplicate for 11 HBM DNA extracts. These included seven DNA extracts randomly selected for repeat processing within a single run (“within-run repeats”); and four DNA extracts randomly selected for repeat processing between the two sequencing runs performed (“between-run repeats”). In addition, the ZymoBIOMICS™ Microbial Community DNA Standard (Catalogue no. D6305, Zymo Research Corp., Irvine, CA, USA) was used to assess library preparation, sequencing reproducibility, and bias.

### 2.5. 16S Ribosomal Ribonucleic Acid (rRNA) Gene Amplicon Library Preparation and Sequencing

Previously published primers [18] (with slight modifications [19]), PCR conditions, and library preparation steps were followed to generate 16S rRNA gene libraries [19]. Libraries were constructed using a two-step amplification approach described by Wu and colleagues [20]. In the first PCR reaction, the hypervariable V4 region of the 16S rRNA gene was amplified using primers (515Fshort)-5′GTGCCAGCHGCYGCGGT3′ and (806Rshort)-5′GGACTACNNGGGTNTCTAAT3′, respectively. Each 25.25 µL PCR reaction contained 12.5 µL 2X MyTaq™ HS Mix (BIO-25046), 2 µL each of 0.8 µM forward and reverse primers, 1 µL of MilliQ water, 0.75µL dimethyl sulphoxide (Sigma-Aldrich^®^, MO, USA), and 7 µL bacterial genomic DNA. PCR was carried out under the following conditions: 95 °C for 3 min; 10 cycles of 95 °C for 30 s, 50 °C for 30 s, and 72 °C for 1 s; and, finally, 72 °C for 5 min. Amplicon product (7 µL) from the first PCR reaction was used as template in the second PCR reaction. The second PCR reaction mixture was the same as the first except for the use of modified reverse primers. Reverse primers were modified at the 3′ end with Illumina adapter sequences and unique index barcodes for each sample. The use of index sequences allowed multiplexing of samples in a single sequencing run [18]. The PCR conditions for the second PCR were the same as for the first PCR but run for 30 cycles.

Amplicon products were cleaned with the Agencourt AMPure system (Beckman Coulter, UK) using an Agencourt SPRIPlate 96 super Magnet Plate. The QuantiFluor™ dsDNA System (Promega, Madison, WI, USA) was used to quantify the cleaned amplicons. The integrity of the cleaned amplicons was assessed by gel electrophoresis. Amplicons were normalized by pooling the different samples at 100 ng each. The pooled library was purified using the Agencourt AMPure system and extracted from a 1.5% agarose gel following gel electrophoresis (30 min at 35 V; 45 min at 40 V; and finally, 3 h 30 min at 70 V). Excision of the pooled 16S library was followed by purification using QIAquick Gel Extraction Kit (Qiagen, Valencia, CA, United States) in accordance with manufacturer’s protocol. The elution buffer, Tris-EDTA (pH 8.0), was heated at 70 °C to improve amplicon recovery (step 13). Finally, the Qubit^®^ dsDNA BR Assay Kit was used for quantification of the pooled library.

Sequencing was carried out at the Centre for Proteomic and Genomic Research, Cape Town, South Africa. Quality control pre-sequencing steps included quantification of adapter-ligated dsDNA using the KAPA Library Quantification Kit (Illumina^®^) (KAPA Biosystems, MA, USA) and analysis of the fragment size of the pooled library using the Agilent High-Sensitivity (HS) DNA Kit (Agilent Technologies, CA, USA). The library pool was diluted to 5.5 pM for sequencing, and the internal sequencing control (PhiX control V3, Illumina, CA, USA) was spiked into the diluted library at 15% (v/v), in accordance with the manufacturer’s instructions [21]. The first and second library pool had 352 and 202 samples multiplexed per Illumina run. The pooled 16S library was paired-end sequenced on the Illumina MiSeq^®^ system using the MiSeq^®^ Reagent v3 kit (600 cycles) (Illumina, CA, USA).

### 2.6. Processing of 16S rRNA Gene Sequences

The 16S rRNA gene sequences were processed using an in-house pipeline. Briefly, the sequencing quality of FASTQ files was assessed using FastQC and MultiQC packages [22,23]. Forward and reverse sequences were then merged using UPARSE [24], allowing 3 mismatches in overlaps (uparse_merge_fastq, fastq_maxdiff set to 3), followed by quality filtering using uparse_filter_fastq fastq_filter (sequences truncated to 250bp). Reads with a maximum expected error >0.1 were discarded (fastq_maxee set to 0.1). The USEARCH10 [25] sortbysize command was used to dereplicate and select sequences occurring more than once. Clustering of sequences into operational taxonomic units (OTUs) (with a clustering radius of 3) was performed with USEARCH10. The USEARCH10_uchime2_ref tool was used to detect and remove chimeras, and OTU counts were obtained using USEARCH10 usearch-global.

In silico correction for contamination of biological HBM samples was carried out using a procedure to control for “over-compensation” during contaminant removal (Supplementary Figure S1). Firstly, *M. smegmatis* sequences were removed from the “*M. smegmatis*-spiked-NTC” controls. Background sequences present in the *M. smegmatis*-spiked-NTC controls after the removal of *M. smegmatis* sequences were screened against biological sample sequences by aligning biological sample sequences to background sequences at 100% identity using align_seq.py, based on PyNAST [26] algorithm and uclust [25] (Supplementary Figure S1D). The average number of reads was calculated for each of the “background sequences” across *M. smegmatis*-spiked-NTCs, and the corresponding number of reads was thereafter removed from the biological HBM samples (Supplementary Figure S1E). Further processing of data was performed using Quantitative Insights Into Microbial Ecology (QIIME) 1.9.0 suite of software tools [27]. Taxonomy was assigned to representative sequences of the OTUs using assign_taxonomy.py (method set to the Ribosomal Database Project (RDP)) classifier [26], and identity was set to 97% with the SILVA 132 release database [28]. Sequence alignment and filtering was performed with align_seqs.py (97% identity) and filter_alignment.py, while construction of a phylogenetic tree was done using the make_phylogeny.py script.

Chao1 species richness [29] was used to estimate species richness, and a rarefaction plot of Shannon diversity against sequencing depth was generated using alpha_rarefaction.py in QIIME 1.9.0. Samples with <1000 reads were removed from further analysis as the rarefaction curve plateaued at a sequence depth of 1000 read counts. The core bacteriome, defined as the OTUs that were present in at least 80% of the samples, was determined with QIIME with the script “compute_core_microbiome.py”. Nextflow [30] was used to loop the entire processing workflow. The 16S rRNA gene sequencing datasets used in this study are stored in the National Center for Biotechnology Information (NCBI) Sequence Read Archive (SRA) repository (BioProject PRJNA520889).

### 2.7. Statistical Analyses

Statistical analysis and graphical illustrations of the data (barplots, boxplots, dendograms) were generated in R statistical package (version 3.4.4) [31] and R studio 1.1.456, respectively. Alpha diversity was calculated using the Shannon–Weaver index [32]. Analysis of variance (Type II test) [33] was used to test the difference in alpha diversity between groups, while error estimates were based on Pearson residuals.

Agglomerative clustering was performed for all OTUs with relative abundance of >0.5% and was generated by complete linkage hierarchical clustering [34] using the [hclust] function [35]. This hierarchical clustering method is based on the Bray–Curtis dissimilarity index [36] of the R *vegan* package [37]. To determine the relative abundances of bacterial profiles, the operational taxonomic unit (OTU) table (clustered at 97% sequence similarity) was transformed from count data to compositional data [38]. The hierarchical clustering tree was cut at a height of 0.8 to cluster samples into groups based on relative abundance of bacteria in HBM samples at genus level. The optimal number of clusters was determined using the Calinski–Harabasz index [39] and validated by the silhouette width index [40].

Log–ratio biplots using a Bayesian prior technique for adjustments of zero counts were made as previously described [41], and lambda-scaling was employed to ensure evenness in the “total spread” of the data sets [42]. Box-plots [43] were used for visualization of distribution of the data.

A co-occurrence network was constructed using the R network package with centered log–ratio data [44]. The cut-off was set to 0.28 to generate the network for each of the different clusters and for all samples, using bacteria with genus-level abundances >0.5%. A bivariate correlation analysis for the 16 most abundant genera was performed using Pearson correlation.

Permutational multivariate analysis of variance using distance matrices (PERMANOVA) via “adonis” in the R package *vegan* [45,46] with Aitchison’s distance [47,48] in the R package *robCompositions* [49] was used to test the effects of potential influencing factors on the composition and diversity of HBM bacteriome. ADONIS was performed with 999 x permutations and method “bray”. The Benjamini–Hochberg method for multiple correction was used to correct all *p*-values, set at a 5% significance level, by the false discovery rate (FDR) [50]. All OTUs irrespective of their abundance at each taxonomy level were tested in the model, and a multivariate *p*-value was generated for the covariates. Linear discriminant analysis (LDA) effect size (LEfSe) with default parameters [51] was used to assess taxa that differed between groups, for any covariate significant by ADONIS.

The Beck depression inventory (BDI-II) is a self-report measure of depressive symptoms that assesses key symptoms of depression between 0 (absence of symptom) and 3 (severe, often with functional impairment). Individual items are summed, and scores of ≥20 is indicative of moderate/severe depression [52,53]. The self-regulation questionnaire (SRQ) is a WHO-endorsed self-report measure assessing psychological distress, including symptoms of depressive and anxiety disorders in which individual items are scored according to whether or not the symptom is present. SRQ scores ≥8 is indicative of “high risk” participants [54,55]. PTSD was measured by the modified posttraumatic stress disorder symptom scale (MPSS) [56], which is a self-report measure with scale including items which assess 3 symptom clusters for PTSD: re-experiencing, avoidance/emotional numbing, and increased arousal. Items assessing the re-experiencing symptom cluster were scored as “above threshold” if their sum totaled ≥1; avoidance/emotional numbing ≥3; and increased arousal ≥2. Participants who scored above threshold across all three symptom clusters and reported symptom duration of at least 1 month (scored ≥1 for item 18) were classified as “suspected PTSD cases”. An intimate partner violence (IPV) questionnaire was used to assess recent exposure to physical, emotional, and sexual IPV as previously described [57]. The psychosocial variables described above were collected at the 28–32-week antenatal visit.

The alcohol, smoking, and substance involvement screening test (ASSIST) was used to assess maternal alcohol consumption based on a scoring system as previously described [57]. Scores of 0–10 for alcohol indicate that a participant is at a low risk for substance-related health problems.

Infant length was measured in centimeters to the nearest completed 0.5 cm, and weight was measured in kilograms to the nearest completed 10 g at the hospital as previously described [58].

A coefficient of multiple determination was generated using linear regression analysis [59] to determine the reproducibility of the eleven DNA extracts which were processed in duplicate. The total read count of each OTU in a sample was compared with the total read count of each OTU in its duplicate set.

## 3. Results

### 3.1. Participant Characteristics

HBM samples were collected from 554 mothers at 6–10 weeks (median 7.5 weeks) postpartum. Maternal characteristics can be found as Supplementary Table S1. The median maternal age at enrolment was 25.3 years. The median maternal weight and body mass index (BMI) at time of HBM collection was 63.7kg and 25 kg/m^2^, respectively. The majority of the mothers were unemployed, and only 13% of participants had a household income of >ZAR5000/month (>360 USD/month). HIV prevalence was higher at the Mbekweni study site (18.4% of mothers) compared to TC Newman (2% of mothers). Smoking status, as measured by urine cotinine levels at the 28–32-week antenatal visit, classified 13% of women at Mbekweni as active smokers compared to 52% of mothers at TC Newman. At the 6–10-week postpartum study visit, only 53% of women were exclusively breastfeeding their babies. Most of the women delivered their infants vaginally (81%). Maternal posttraumatic stress disorder (PTSD) was documented in 8% of mothers, while 25% and 22% of women were above the Beck depression inventory (BDI) and self-regulation questionnaire (SRQ) threshold, respectively. The median household size was 5 people across both sites. Twenty-three percentage (23%) of infants were delivered pre-term (Supplementary Table S1).

### 3.2. Sequencing Results and OTU Analysis

A total of 16,835,376 high-quality raw paired-end reads were obtained. The total number of postfiltered reads was 4,865,561. The median and mean sequence read count per sample was 8007 and 8782.6 (range 48–40,919), respectively. A rarefaction curve of Shannon diversity plateaued at a sequence depth of 1000 read counts (Supplementary Figure S2), therefore 32 samples with sequence read counts <1000 were removed from further analysis. USEARCH9 mapping of sequences revealed that the sequence reads clustered into 2284 OTUs. The OTUs were classified into 58 phyla, 133 classes, 263 orders, 596 families, and 1300 genera.

### 3.3. Profiling of Human Breast Milk Bacteriome

**Figure 1:**
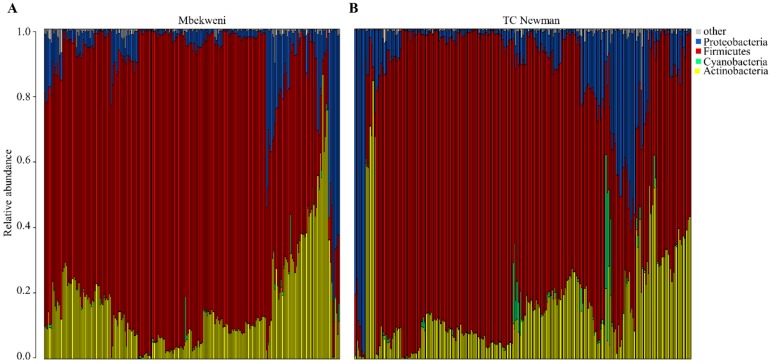
Complete linkage unsupervised hierarchical clustering of relative abundances of human breast milk (HBM) bacterial phyla. Bar-plot of relative abundances of HBM bacteria genera at (**A**) Mbekweni and (**B**) TC Newman study sites. Each bar represents a mother’s HBM bacteriome profile and each colored box, a bacterial phylum. Phyla with less than 0.5% abundance in a given sample are grouped together, herein referred to as “other” (grey boxes).

HBM bacterial profiles revealed diverse bacterial communities. Four phyla had mean relative abundances >0.5%: Firmicutes (mean relative abundance (MRA): 72%), Actinobacteria (16%), Proteobacteria (10%), and Cyanobacteria (0.9%) (Figure 1). At the genus level, 16 genera had MRAs >0.5%, of which *Streptococcus* (49%), *Staphylococcus* (18%), and *Rothia* (6%) were most abundant (Supplementary Figure S3). A core bacteriome consisting of 14 OTUs from 9 genera, *Corynebacterium, Streptococcus, Staphylococcus, Rothia, Veillonella, Gemella, Acinetobacter, Micrococcus*, and a genus belonging to the Enterobacteriaceae family, was observed (these genera were all present in HBM samples from >80% of women) (Supplementary Table S2).

### 3.4. Breast Milk Bacteriome Profiles Segregate into Three Major Clusters

**Figure 2:**
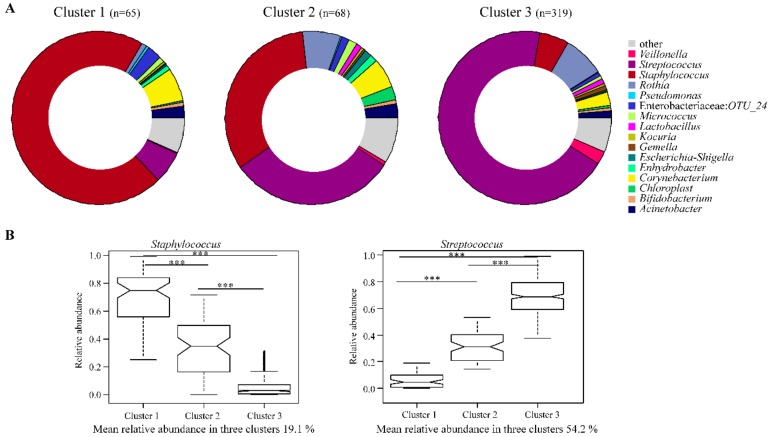
Human breast milk (HBM) bacterial profiles of the three clusters in which samples were grouped at genus level. (**A**) Pie charts show the mean relative abundances in each cluster at the genus level (for genera with abundance >0.05%). (**B**) Notched box plots show the mean relative abundance of the predominant bacteria genera in each cluster. The notched box signifies the 75% (upper) and 25% (lower) quartile, showing the distribution of 50% of the samples. The median is represented by the line inside the box plot, and the notch shows the 95% confidence interval for the median. The whiskers (top and bottom) represent the maximum and minimum values. Outliers, which are beyond 1.5 times the interquartile range above the maximum value and below the minimum value, are shown with open circles. (^∗∗∗^*p* < 0.001).

Hierarchical complete linkage unsupervised clustering based on Bray–Curtis dissimilarities at the genus level resulted in an eleven-cluster best fit model. Clusters 4–11 however, had very few participants and were therefore excluded from further analysis (Supplementary Figure S4). *Streptococcus* and/or *Staphylococcus* were responsible for the formation of the three dominant clusters in our study population. Cluster 1 showed the highest abundance of *Staphylococcus* spp., cluster 2 was dominated by relatively equal proportions of both *Streptococcus* spp. and *Staphylococcus* spp., while cluster 3 showed the highest relative abundance of *Streptococcus* spp. (Figure 2). Similar numbers of HBM samples were found in clusters 1 and 2, with the majority in cluster 3 (Figure 2A).

Bacterial profiles of many of the most abundant taxa also differed between the cluster groups. At phylum level, cluster 2 showed the highest abundance of Actinobacteria and Proteobacteria but lowest abundance of Firmicutes. Cluster 3, on the other hand, showed the highest abundance of Firmicutes but lowest abundance of Proteobacteria (Supplementary Table S3). At genus level, cluster 1 showed the highest abundance of OTU_24 (belonging to the family Enterobacteriaceae) and *Corynebacterium,* with the lowest abundance of *Veillonella* and *Rothia;* cluster 2 showed the highest abundance of *Acinetobacter* and the LAB-*Lactobacillus* and *Bifidobacterium*; cluster 3 showed the highest abundance of *Veillonella* and *Rothia* but lowest abundance of *Corynebacterium, OTU_24* and *Acinetobacter* (Figure 2 and Supplementary Table S3).

**Figure 3:**
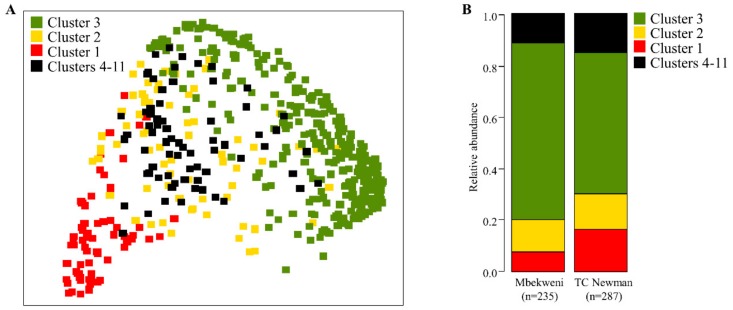
Human breast milk (HBM) bacteriome profiles colored by clusters. (**A**) Principal coordinate analysis (PCoA) of Bray–Curtis distance matrices of bacteria genera from all 522 HBM samples in the study. (**B**) Bar plots showing the proportion of different clusters detected from each study site.

Furthermore, exploration of β-diversity using principal coordinate analysis (PCoA) revealed distinct separation of HBM samples based on the three dominant bacterial profile cluster groups (Figure 3A). PERMANOVA “adonis” analysis identified significant dissimilarity in bacterial composition between the clusters at order, family, and genus taxonomy levels (*p* ≤ 0.001). Cluster 3 was common at both study sites but more prevalent at Mbekweni, while cluster 1 was more prevalent at TC Newman (*p* = 0.018) (Figure 3B).

### 3.5. Alpha Diversity of Bacterial Communities within the DCHS Cohort Study

**Figure 4:**
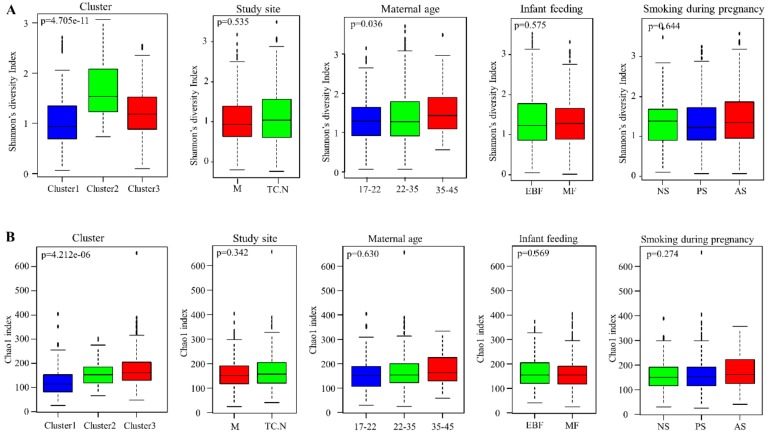
Alpha diversity indices of human breast milk bacteriome profiles. (**A**) Shannon diversity index, and (**B**) Chao1 index of HBM bacteriome profiles of participants based on cluster group, study site, maternal age (in years), infant feeding at 6–10 weeks, and smoking during pregnancy. M = Mbekweni, TCN = TC Newman, EBF = exclusive breastfeeding, MF = mixed feeding, NS = non-smoker, PS = passive smoker, AS = active smoker.

Of the 26 covariates tested in this study (Supplementary Table S1), only the phenotypic cluster groups and infant birth length showed significant differences in both alpha diversity measures (Shannon diversity and Chao1 index). Figure 4 shows the alpha diversity matrices for 5 selected covariates. For phenotypic cluster groups, cluster 1 had the lowest diversity by both measures, while cluster 2 had the highest Shannon diversity and cluster 3 had the highest Chao1 species richness (Figure 4). To analyze the association of infant birth length with HBM bacterial diversity, samples were stratified into three groups: mothers with infant birth length of 0–40, 40–48, and 48–60 cm. HBM from mothers with infant birth length 48–60 cm had the lowest bacterial diversity by both measures (Supplementary Table S4). A significant difference in Shannon diversity was observed based on maternal age in all samples and at the Mbekweni study site, with older mothers (ages ≥35) having a higher Shannon diversity index (*p* < 0.05) (Figure 4 and Supplementary Table S4).

### 3.6. Human Breast Milk Bacterial Profiles in Relation to Demographic, Socio-Economic, and Psychosocial Variables

**Figure 5:**
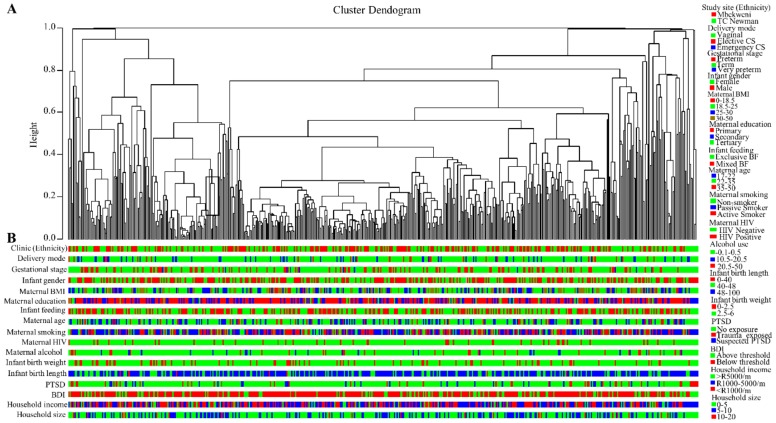
Complete linkage unsupervised hierarchical clustering of human breast milk (HBM) bacterial profiles. (**A**) Dendogram of bacterial profiles present in 522 HBM samples at genus level. (**B**) Horizontal colored bars below the dendogram summarize covariate data for each sample.

**Figure 6:**
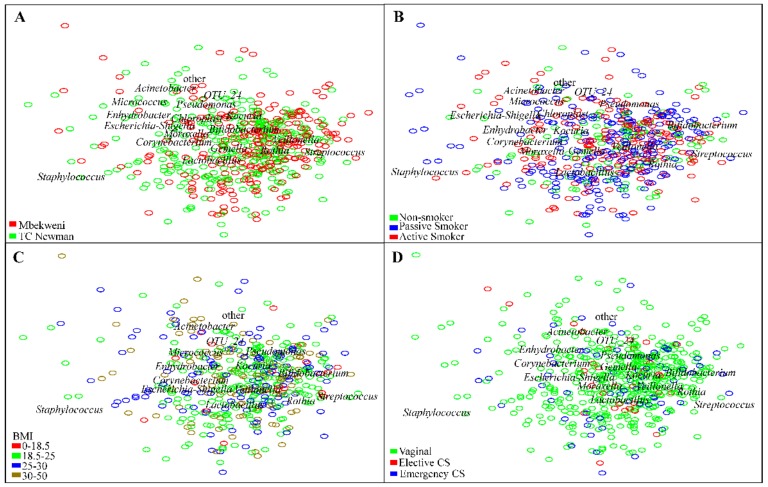
Log–ratio biplot of human breast milk bacterial abundances at the genus level. Samples are colored according to (**A**) study site, (**B**) maternal smoking, (**C**) body mass index (BMI), and (**D**) delivery mode. Each sample is represented by a circle and is colored based on group type. Samples (circles) that cluster together are similar in bacterial composition and abundance.

Beta-diversity clustering at the genus level of all HBM samples based on Bray–Curtis dissimilarity index showed no clear association between bacterial profiles and the different covariates studied (Figure 5). We also visualized bacterial diversity at the genus level for genera with abundances >0.5% using log–ratio biplots. Clustering was not observed for any covariates studied (Figure 6 and Supplementary Figure S5). Figure 6 shows the results for a selection of 4 covariates (study site, maternal smoking, maternal BMI, and mode of delivery). Mothers with elective caesarean section delivery had bacterial profiles with less clustering towards *Staphylococcus* (Figure 6).

Associations between bacterial profiles and socio-demographic and psychosocial characteristics were also investigated at different taxonomy levels using PERMANOVA. At the phylum, order, family, and genus levels, only study site (which was also a marker for ethnicity) was associated with significant dissimilarity of the HBM bacterial communities (ANOVA, *p* < 0.05). There were no statistically significant differences between HBM bacterial profiles in relation to the remaining covariates (Table 1). LEfSe was performed to further explore differences in specific bacterial taxa in relation to study site. In line with the prevalence of the different cluster groups between study sites, HBM samples from Mbekweni had significantly higher relative abundance of the genus *Streptococcus*, while samples from TC Newman had significantly higher relative abundances of the genera *Staphylococcus*, *Acinetobacter*, and *Escherichia_Shigella* (Supplementary Figure S6A).

### 3.7. Co-occurrence Networks in Human Breast Milk Bacterial Communities

**Figure 7:**
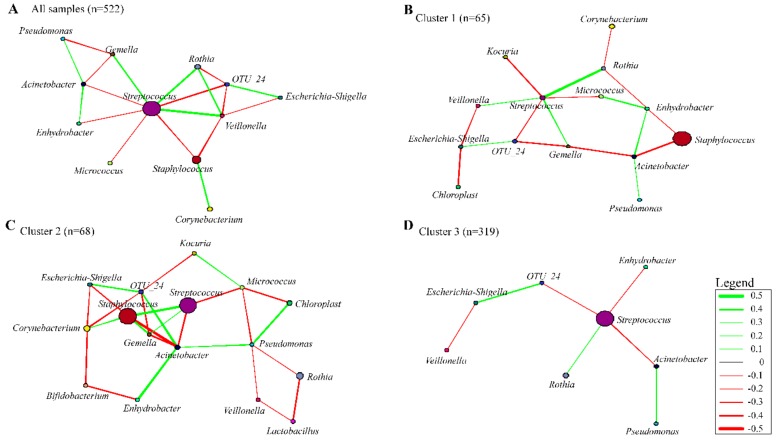
Bacterial networks within the human breast milk bacteriome community based on correlation between relative abundances. Figures show correlations between predominant bacteria in (**A**) all HBM samples, (**B**) cluster 1, (**C**) cluster 2, and (**D**) cluster 3. Nodes and node sizes represent bacterial genera and their relative abundances, respectively. Green and red lines represent positive and negative correlations respectively between the bacteria, with the thickness of the line indicating the degree of correlation.

Bacterial correlations within the HBM bacteriome were explored, since bacteria cohabit ecological niches, and interactions between bacterial species may be present [60]. We constructed a network for all HBM samples, and across the 3 main clusters based on Pearson’s correlations of relative abundance (−0.28 >|r|<0.28) at genus level (Figure 7 and Supplementary Table S5). The network of all HBM samples contained 12 nodes (bacterial genera) and 19 edges (interconnecting lines denoting correlations) (Figure 7A). Bacterial correlations within each cluster group were similar except for *Streptococcus* spp. and *Staphylococcus* spp. While a negative correlation was observed between these two genera in a co-occurrence network involving all samples, a positive interaction was observed in cluster 2, where the relative abundances of *Streptococcus* spp. and *Staphylococcus* spp. were similar.

Within the Firmicutes phylum, the abundance of *Staphylococcus* spp. was positively correlated with *Corynebacterium* spp. (both common commensals of the skin) but was negatively correlated with *Streptococcus* spp. and *Veillonella* spp. *Streptococcus* spp. abundance, on the other hand, was positively correlated with other members of the oral flora, *Veillonella* spp., *Gemella* spp., and *Rothia* spp. and negatively correlated with the Proteobacteria (*Acinetobacter* spp., and *Enhydrobacter* spp.)*,* and with skin commensals such as *Micrococcus* spp. and *Staphylococcus* spp. A positive correlation was observed between *Pseudomonas* spp., *Acinetobacter* spp., and *Enhydrobacter* spp., all members of the order Pseudomonadales.

### 3.8. Reproducibility of Bacterial Profiling

The bacterial composition of each sample and its repeat was evaluated to test for reproducibility. Multiple R-square values of >0.97 for both within-run and between-run repeats showed excellent reproducibility (Supplementary Figure S7). Hierarchical clustering also showed that replicate samples clustered together (Supplementary Figure S8).

## 4. Discussion

In this study, the largest of its kind to date, we described the composition of the HBM bacteriome in samples from mothers living in South Africa. We identified a core bacteriome consisting of 9 bacterial genera present in >80% of samples. We also showed that there are three major HBM bacteriome profiles, distinguished by the relative abundances of the genera *Streptococcus* and *Staphylococcus*, and that bacteria which are commonly found as part of the skin flora correlate in relative abundance in HBM, as do bacteria which are part of the oral microflora. We were able to demonstrate an association between study site (a proxy for ethnicity in this study), infant birth length and maternal age, and the composition of the HBM bacteriome.

Previous studies of the HBM bacteriome have revealed a diverse bacterial population, including gram positive (*Corynebacterium* spp., *Lactobacillus* spp., *Bifidobacterium* spp., *Staphylococcus* spp.) and gram negative (*Pseudomonas* spp., *Veillonella* spp.) bacteria [7,9,61,62]. The most abundant bacterial phyla in our study were Firmicutes and Actinobacteria, in contrast to several other studies [8,9,63,64], which described a predominance of Proteobacteria and Firmicutes. Our findings are similar, however, to that of Williams et al. [13], who showed similar relative abundances at the phylum level in HBM from mothers in the USA. Many factors, including geographical region (which influences diet and cultural practices) [65], lactational stage, and maternal health, may influence the HBM bacterial community, with considerable diversity reported between individuals [5,63]. Methodological differences including sample processing and analytical techniques may also account for differences between studies.

At the bacterial genus level, *Streptococcus* spp. and *Staphylococcus* spp. were most abundant, in line with previous studies [7,8,9,63,66,67] and a recent systematic review [68]. Other abundant genera included *Rothia* spp., *Corynebacteria* spp. and *Acinetobacter* spp. Of note, the relative abundance of *Acinetobacter* spp. observed in our cohort (2.2%) was low. When comparing our findings to those published previously, we found that studies with sample collection protocols similar to ours identified *Acinetobacter* spp. proportions comparable to those in our study [66]. By contrast, proportions of *Acinetobacter* spp. were higher (32%) when the sample collection protocol omitted cleaning of breast skin prior to collection and discarding of the first few drops of milk during the collection procedure [66].

*Bifidobacterium* spp. and *Lactobacillus* spp. (lactic acid bacteria (LAB)) were detected at low relative abundances (mean relative abundances of 1.0% and 0.9%) but detected in 72% and 76% of women in our study. Previous studies using quantitative polymerase chain reaction (qPCR) estimated the absolute abundance of *Lactobacillus* spp. and *Bifidobacterium* spp. as 10^3^–10^4^ cells/mL and 10^2^–10^5^ cells/mL, respectively [4,69,70]; however, we were not able to determine absolute abundance using 16S rRNA gene amplicon sequencing. LAB have been identified as probiotics which colonize the infant gut even at a relatively small dose, and function to competitively exclude pathogens [71,72]. Studies have suggested that HBM is an important source of *Bifidobacterium* for the infant gut. In support of this, it has been shown that *Bifidobacterium* dominates the gut of a breastfed full-term infant as early as the first 3–6 days of life and makes up 60%–90% of the total bacteriome of the infant gut [73]. Furthermore, identical strains of *Bifidobacterium breve* and *Lactobacillus plantarum* have been isolated from HBM and infant feces in a mother–infant pair, confirming vertical transmission [9].

Our study confirmed the existence of a shared and conserved “core” bacteriome in HBM, which is consistent with previous studies [7,8,9,64]. The core bacteriome identified from HBM samples assessed in our study included eight bacterial genera at 80% prevalence. *Bifidobacterium* and *Lactobacillus* were not part of the core bacteriome in our study, in contrast to a previous study in Ireland [9].

Further investigation into HBM bacterial profiles using clustering analysis revealed three major dominant profiles present in our samples, determined by the relative abundances of *Streptococcus* spp. and/or *Staphylococcus* spp. Cluster 1, dominated by *Staphylococcus* spp., was associated with a less diverse bacteriome. A previous study in Taiwan and mainland China similarly described clustering of bacterial profiles based on the dominance of *Streptococcaceae*, and *Staphylococcaceae*, but at the family taxonomy level [63]. In contrast to our study, however, *Pseudomonadaceae* contributed to the third cluster [63].

### 4.1. Bacterial Interactions within the Human Breast Milk Bacterial Community

We explored the correlation between bacterial genera, based on relative abundance. We showed a positive relationship between the two skin commensals, *Staphylococcus* spp. and *Corynebacterium* spp., in line with previous findings by Ma et al. (2015) [74]. Recently, an “evil alliance” was proposed to occur between these two genera, as both have been implicated as the cause of mastitis in breastfeeding women [74,75]. *Staphylococcus epidermidis* and *Staphylococcus aureus* are major causes of infectious mastitis, a major reason of premature cessation of breastfeeding among lactating women [2]. *Corynebacterium* spp. has been implicated as the third most prevalent bacterial group causing infectious mastitis [75].

*Veillonella* spp., *Gemella* spp., *Rothia* spp. and *Streptococcus* spp., which are commensals of the oral cavity, demonstrated positive correlations. A similar positive correlation was also observed between *Pseudomonas* spp., *Acinetobacter* spp. and *Enhydrobacter* spp., which are all members of the order Pseudomonadales and live in the nasal cavities.

A negative correlation was observed between the abundances of *Acinetobacter* spp. and *Staphylococcus* spp. Unlike in a previous study in Taiwan and Mainland China [63], correlations between *Streptococcus* spp. and *Staphylococcus* spp. varied between cluster groups, being negative for clusters 1 and 3, but positive for cluster 2 where both bacterial genera had high relative abundances.

### 4.2. Impact of Maternal and Infant Factors on Human Breast Milk Bacterial Profiles

We studied a broad range of potential influencing factors of the HBM bacteriome but showed no association of maternal, socio-economic, and psychosocial variables with the HBM bacteriome, apart from maternal age, infant birth length, and study site (which almost completely correlated with ethnicity). Our findings are similar to those of Urbaniak and colleagues (2016), who found that HBM bacterial profiles did not differ significantly based on mode of delivery, gestation or infant gender in a Canadian population [8]. In support of our and Urbaniak and colleagues’ findings, a previous study conducted on HBM samples among Chinese women (*n* = 90) and a longitudinal study conducted among American women (*n* = 104) also found that the mode of delivery had no influence on HBM bacteriome [13,66].

In contrast to these reports, two recent studies show separation of HBM bacterial profiles on principal component analysis of beta diversity in relation to mode of delivery [64,76]. Both studies, however, had relatively small sample sizes (12 and 84 participants). In our study, we observed separation of HBM bacterial profiles of women who underwent elective caesarean section from HBM profiles of women who underwent either vaginal or emergency caesarean section delivery in a log–ratio biplot with less clustering around *Staphylococcus*; however, this association did not show statistical significance on PERMANOVA testing. Kumar et al. (2016) showed that while delivery mode was not associated with HBM bacterial profiles among South African and Finnish women, Chinese and Spanish women showed different bacterial profiles based on mode of delivery [5]. Li et al. (2017) found no difference in the abundance of bacterial families in HBM based on delivery mode; however, the genus, *Lactobacillus,* differed between the two groups [63]. In another recent study, mode of delivery was associated with bacterial composition of colostrum. Higher abundances of *Pseudomonas* spp., *Staphylococcus* spp., and *Prevotella* spp. were observed in the colostrum of mothers with caesarean section delivery. It is possible that differences in HBM profiles would be most marked in early HBM samples where the hormones involved in the delivery process still have an impact.

Other factors which have been associated with HBM bacterial profiles include gestational age, maternal age, and maternal BMI. Interestingly, one previous study using qPCR showed differences in HBM composition between preterm and term milk, with higher *Bifidobacterium* spp. and lower *Enterococcus* spp. counts among women who delivered term babies [70]. A more recent study and ours, using 16S rRNA gene sequencing, showed no impact of gestational age on the HBM bacteriome [8]. With focus on infant gender, a previous study reported higher mean relative abundances of *Streptococcus* spp. and lower mean relative abundances of *Staphylococcus* spp. from HBM of mothers with male infants [13], although we did not find any significant association. Contrary to our study, BMI within the normal range was associated with higher proportions of Alphaproteobacteria and Betaproteobacteria at the class level [77]. Maternal age, on the other hand, influenced the diversity of HBM bacterial profiles in our study and previous reports [63,77]. How maternal age or infant sex drives the bacteriome is largely unknown to date.

A study in Taiwan showed no influence of BMI on HBM bacteriome [63], as was found in our study; however, higher *Granulicatella* spp. relative abundance was observed in another study of HBM from overweight and obese mothers [13].

There is increasing awareness of the microbiota–gut–brain axis, with several studies describing an association between the gut microbiota, neuropsychiatric and psychosocial variables in both mouse models and humans [78,79,80]. We, however, did not observe any association between psychosocial variables and the HBM bacteriome. To the best of our knowledge, this is the first study to examine the association of psychosocial variables with HBM bacteriome composition.

In our study, we found an association between study site and the HBM bacteriome. However, since ethnicity was almost completely correlated with study site, we are unable to determine whether these differences were due to ethnicity or other unmeasured factors associated with study site. Li et al. [63] demonstrated similar findings in a study characterizing the HBM bacteriome from various regions within Taiwan and Mainland China [63]. Ethnicity has been found to have an influence on the gut microbiome in several studies [65,81] and could be driven by a range of factors, such as genetic differences or diet [82].

Strengths of our study include large sample size, consistent methods for sample collection, processing and sequencing, detailed metadata collection, a reproducible sequencing pipeline, and robust multivariate statistical analysis. The major limitation of our study was the use of a single sampling time point, which precludes us from studying intra-individual variability or exploring changes in bacterial profiles and associated risk factors over time.

## 5. Conclusions

We used 16S rRNA gene sequencing to characterize the HBM bacteriome of a large cohort of mothers living in South Africa. We showed that the HBM bacteriome was dominated at the phylum level by Firmicutes and Actinobacteria, and at the genus level by *Staphylococcus, Streptococcus*, and *Rothia.* We identified three major microbiome profile groups, defined by the relative abundances of *Staphylococcus* spp. and *Streptococcus* spp. We found little evidence of the association of various socio-economic or psychosocial variables with the HBM bacteriome, but we showed that maternal age, infant birth length, and study site were associated with composition of the HBM bacteriome.

## Figures and Tables

**Table 1:** Permutational multivariate analysis of variance (PERMANOVA) analyses of HBM bacteriome and its association with maternal demographic and psychosocial factors.

Covariates	Phylum (*p*-Value)	Class	Order	Family (*p*-Value)	Genus (*p*-Value)
		(*p*-Value)	(*p*-Value)		
Study site (Ethnicity)	0.041 *	0.0628	0.0036 *	0.004 *	0.0028 *
Mode of delivery	0.9756	0.9492	0.8276	0.8358	0.793
Gestational age	0.1266	0.126	0.944	0.3424	0.3538
Infant gender	0.7818	0.8142	0.3084	0.9608	0.7898
Infant feeding options	0.1716	0.2244	0.9502	0.2888	0.4662
Maternal education	0.275	0.3486	0.2202	0.189	0.1734
Maternal employment	0.7434	0.7192	0.2346	0.6372	0.7988
Maternal BMI	0.3886	0.3806	0.6206	0.714	0.5788
Infant birth weight	0.836	0.933	0.6886	0.719	0.7824
Infant birth length	0.9876	0.971	0.9006	0.895	0.9414
Maternal age	0.3286	0.3986	0.8976	0.641	0.7316
Dwelling type	0.5846	0.4892	0.6306	0.136	0.1158
Marital status	0.4194	0.4406	0.1362	0.7608	0.7778
Household income	0.6062	0.6988	0.663	0.0922	0.0988
Maternal HIV status	0.7238	0.6582	0.0836	0.9242	0.9252
Antibiotics	0.4178	0.4446	0.951	0.3334	0.2128
Household size	0.559	0.4248	0.3374	0.9198	0.8276
Maternal smoking	0.867	0.8698	0.837	0.7986	0.7448
Alcohol score	0.1216	0.0974	0.8012	0.2018	0.8706
IPV-emotional	0.9198	0.9132	0.806	0.8394	0.3256
IPV-physical	0.2504	0.2432	0.1708	0.6604	0.817
IPV-sexual	0.7438	0.8552	0.8676	0.2556	0.8092
PTSD	0.847	0.6846	0.724	0.3232	0.1342
BDI score	0.127	0.876	0.2598	0.3168	0.3246
SRQ	0.7408	0.5376	0.2576	0.8204	0.3012

EBF = exclusive breastfeeding; BMI = body mass index; IPV = induced partner violence; PTSD = posttraumatic stress disorder; BDI = Beck depression inventory; SRQ = self-regulation questionnaire; smoking status was based on cotinine levels measured during an antenatal visit. Cotinine levels ≥500 (active smoker); cotinine levels >10<500 (passive smoker); cotinine levels ≤10 (non-smoker). *p*-values were generated by multivariable PERMANOVA analyses and adjusted using Benjamini–Hochberg’s false discovery rate. Significant results (*p* < 0.05) are labeled with asterisks.

## Supplementary Materials

The following are available online at https://www.mdpi.com/2072-6643/11/6/1390/s1, Figure S1: In-silico de-contamination of biological human breast milk samples, Figure S2: Alpha rarefaction curve relating the sequencing depth to Shannon diversity index of all sequenced breast milk samples, Figure S3: Bar-plot of relative abundances of bacteria genera, Figure S4: Dendogram of relative abundances of bacteria in human breast milk (HBM) samples at genus level, Figure S5: Log ratio biplot of human breast milk bacterial abundances at the genus level, Figure S6: Linear discriminant analysis (LDA) effect size (LEFSe) plots showing differences between the human breast milk (HBM) bacteriome of participants based on study site, Figure S7: 16S rRNA gene sequencing reproducibility, Figure S8: Bar-plot of relative abundances of bacteria genera in human breast milk (HBM) samples and their technical repeats, Table S1: Demographic and clinical characteristics of mothers and their infants, Table S2: Core taxa in human breast milk bacteriome, Table S3: Mean relative abundance of dominant phyla and genera (>0.5%) across phenotypic clusters in human breast milk (HBM) samples, Table S4: Mean Shannon diversity of human breast milk (HBM) bacteriome profiles across different socio-economic and psychosocial factors, Table S5: Pearson’s correlations between the relative abundances of the predominant bacterial genera in the human breast milk samples.

## References

[1] Pannaraj P.S., Li F., Cerini C., Bender J.M., Yang S., Rollie A., Adisetiyo H., Zabih S., Lincez P.J., Bittinger K. (2017). Association between breast milk bacterial communities and establishment and development of the infant gut microbiome. JAMA Pediatr..

[2] Jimenez E., de Andres J., Manrique M., Pareja-Tobes P., Tobes R., Martinez-Blanch J.F., Codoner F.M., Ramon D., Fernandez L., Rodriguez J.M. (2015). Metagenomic analysis of milk of healthy and mastitis-suffering women. J. Hum. Lact..

[3] Makino H., Kushiro A., Ishikawa E., Muylaert D., Kubota H., Sakai T., Oishi K., Martin R., Amor K.B., Oozeer R. (2011). Transmission of intestinal bifidobacterium longum subsp. Longum strains from mother to infant determined by multilocus sequencing typing and amplified fragment length polymorphism. Appl. Environ. Microbiol..

[4] Martín R., Jiménez E., Heilig H., Fernández L., Marín M.L., Zoetendal E.G., Rodríguez J.M. (2009). Isolation of bifidobacteria from breast milk and assessment of the bifidobacterial population by pcr-denaturing gradient gel electrophoresis and quantitative real-time pcr. Appl. Environ. Microbiol..

[5] Kumar H., du Toit E., Kulkarni A., Aakko J., Linderborg K.M., Zhang Y., Nicol M.P., Isolauri E., Yang B., Collado M.C. (2016). Distinct patterns in human milk microbiota and fatty acid profiles across specific geographic locations. Front. Microbiol..

[6] Cabrera-Rubio R., Mira-Pascual L., Mira A., Collado M.C. (2016). Impact of mode of delivery on the milk microbiota composition of healthy women. J. Dev. Orig. Health Dis..

[7] Hunt K.M., Foster J.A., Forney L.J., Schutte U.M., Beck D.L., Abdo Z., Fox L.K., Williams J.E., McGuire M.K., McGuire M.A. (2011). Characterization of the diversity and temporal stability of bacterial communities in human milk. PLoS ONE.

[8] Urbaniak C., Angelini M., Gloor G.B., Reid G. (2016). Human milk microbiota profiles in relation to birthing method, gestation and infant gender. Microbiome.

[9] Murphy K., Curley D., O’Callaghan T.F., O’Shea C.A., Dempsey E.M., O’Toole P.W., Ross R.P., Ryan C.A., Stanton C. (2017). The composition of human milk and infant faecal microbiota over the first three months of life: A pilot study. Sci. Rep..

[10] Ojo-Okunola A., Nicol M., du Toit E. (2018). Human breast milk bacteriome in health and disease. Nutrients.

[11] Cabrera-Rubio R., Collado M.C., Laitinen K., Salminen S., Isolauri E., Mira A. (2012). The human milk microbiome changes over lactation and is shaped by maternal weight and mode of delivery. Am. J. Clin. Nutr..

[12] Olivares M., Albrecht S., De Palma G., Ferrer M.D., Castillejo G., Schols H.A., Sanz Y. (2015). Human milk composition differs in healthy mothers and mothers with celiac disease. Eur. J. Nutr..

[13] Williams J.E., Carrothers J.M., Lackey K.A., Beatty N.F., York M.A., Brooker S.L., Shafii B., Price W.J., Settles M.L., McGuire M.A. (2017). Human milk microbial community structure is relatively stable and related to variations in macronutrient and micronutrient intakes in healthy lactating women. J. Nutr..

[14] González R., Mandomando I., Fumadó V., Sacoor C., Macete E., Alonso P.L., Menendez C. (2013). Breast milk and gut microbiota in african mothers and infants from an area of high hiv prevalence. PLoS ONE.

[15] Zar H.J., Barnett W., Myer L., Stein D.J., Nicol M.P. (2015). Investigating the early-life determinants of illness in africa: The drakenstein child health study. Thorax.

[16] Lucey J.A., Tamehana M., Singh H., Munro P.A. (1998). Effect of interactions between denatured whey proteins and casein micelles on the formation and rheological properties of acid skim milk gels. J. Dairy Res..

[17] Bogaert D., Keijser B., Huse S., Rossen J., Veenhoven R., van Gils E., Bruin J., Montijn R., Bonten M., Sanders E. (2011). Variability and diversity of nasopharyngeal microbiota in children: A metagenomic analysis. PLoS ONE.

[18] Caporaso J.G., Lauber C.L., Walters W.A., Berg-Lyons D., Lozupone C.A., Turnbaugh P.J., Fierer N., Knight R. (2011). Global patterns of 16s rrna diversity at a depth of millions of sequences per sample. Proc. Natl. Acad. Sci. USA.

[19] Claassen-Weitz S., Gardner-Lubbe S., Nicol P., Botha G., Mounaud S., Shankar J., Nierman W.C., Mulder N., Budree S., Zar H.J. (2018). Hiv-exposure, early life feeding practices and delivery mode impacts on faecal bacterial profiles in a south african birth cohort. Sci. Rep..

[20] Wu L., Wen C., Qin Y., Yin H., Tu Q., Van Nostrand J.D., Yuan T., Yuan M., Deng Y., Zhou J. (2015). Phasing amplicon sequencing on illumina miseq for robust environmental microbial community analysis. BMC Microbiol..

[21] Illumina P. (2014). Miseq® System User Guide.

[22] Andrews S. (2010). Fastqc: A Quality Control Tool for High Throughput Sequence Data. Http://www.Bioinformatics.Babraham.Ac.Uk/projects/fastqc.

[23] Ewels P., Magnusson M., Lundin S., Kaller M. (2016). Multiqc: Summarize analysis results for multiple tools and samples in a single report. Bioinformatics.

[24] Edgar R.C. (2013). Uparse: Highly accurate otu sequences from microbial amplicon reads. Nat. Methods.

[25] Edgar R.C. (2010). Search and clustering orders of magnitude faster than blast. Bioinformatics.

[26] Wang Q., Garrity G.M., Tiedje J.M., Cole J.R. (2007). Naive bayesian classifier for rapid assignment of rrna sequences into the new bacterial taxonomy. Appl. Environ. Microbiol..

[27] Caporaso J.G., Kuczynski J., Stombaugh J., Bittinger K., Bushman F.D., Costello E.K., Fierer N., Pena A.G., Goodrich J.K., Gordon J.I. (2010). Qiime allows analysis of high-throughput community sequencing data. Nat. Methods.

[28] Quast C., Pruesse E., Yilmaz P., Gerken J., Schweer T., Yarza P., Peplies J., Glockner F.O. (2013). The silva ribosomal rna gene database project: Improved data processing and web-based tools. Nucleic Acids Res..

[29] Chao A. (1984). Nonparametric estimation of the number of classes in a population. Scand. J. Stat..

[30] Di Tommaso P., Chatzou M., Floden E.W., Barja P.P., Palumbo E., Notredame C. (2017). Nextflow enables reproducible computational workflows. Nat. Biotechnol..

[31] The R Development Core Team (2018). R: A Language and Environment for Statistical Computing.

[32] Oksanen J., Blanchet F.G., Kindt R., Legendre P., Minchin P.R., O’hara R., Simpson G.L., Solymos P., Stevens M.H.H., Wagner H. (2013). Package ‘vegan’. Community Ecology Package, Version.

[33] Fox J., Weisberg S. (2018). An R Companion to Applied Regression.

[34] Hartigan J.A. (1975). Clustering Algorithms.

[35] Murtagh F. (1985). Multidimensional Clustering Algorithms.

[36] Bray J.R., Curtis J.T. (1957). An ordination of the upland forest communities of southern wisconsin. Ecol. Monogr..

[37] Oksanen J., Blanchet F., Kindt R., Legendre P., Minchin P., O’Hara R., Simpson G., Solymos P., Stevens M., Wagner H. (2017). Vegan: Community Ecology Package.

[38] Anders S., Huber W. (2010). Differential expression analysis for sequence count data. Genome Biol..

[39] Caliński T., Harabasz J. (1974). A dendrite method for cluster analysis. Commun. Stat. Theory Methods.

[40] Rousseeuw P.J. (1987). Silhouettes: A graphical aid to the interpretation and validation of cluster analysis. J. Comput. Appl. Math..

[41] Greenacre M.J. (2010). Biplots in Practice.

[42] Gower J.C., Lubbe S.G., Le Roux N.J. (2011). Understanding Biplots.

[43] Williamson D.F., Parker R.A., Kendrick J.S. (1989). The box plot: A simple visual method to interpret data. Ann. Intern. Med..

[44] Butts C.T. (2008). Network: A package for managing relational data in r. J. Stat. Softw..

[45] Anderson M.J. (2001). A new method for non-parametric multivariate analysis of variance. Austral Ecol..

[46] Excoffier L., Smouse P.E., Quattro J.M. (1992). Analysis of molecular variance inferred from metric distances among DNA haplotypes: Application to human mitochondrial DNA restriction data. Genetics.

[47] Pawlowsky-Glahn V., Egozcue J.J., Tolosana-Delgado R. (2015). Modeling and Analysis of Compositional Data.

[48] Aitchison J., Barceló-Vidal C., Martín-Fernández J., Pawlowsky-Glahn V. (2000). Logratio analysis and compositional distance. Math. Geol..

[49] Templ M., Hron K., Filzmoser P. (2011). Robcompositions: An R-Package for Robust Statistical Analysis of Compositional Data.

[50] Benjamini Y., Hochberg Y. (1995). Controlling the false discovery rate: A practical and powerful approach to multiple testing. J. R. Stat. Soc. Ser. B (Methodol.).

[51] Segata N., Izard J., Waldron L., Gevers D., Miropolsky L., Garrett W.S., Huttenhower C. (2011). Metagenomic biomarker discovery and explanation. Genome Biol..

[52] Lasa L., Ayuso-Mateos J., Vazquez-Barquero J., Dıez-Manrique F., Dowrick C. (2000). The use of the beck depression inventory to screen for depression in the general population: A preliminary analysis. J. Affect. Disord..

[53] Beck A.T., Steer R.A., Brown G.K. (1996). Beck depression inventory-ii. San Antonio.

[54] Beusenberg M., Orley J.H., World Health Organization (1994). A User’s Guide to the Self Reporting Questionnaire (srq).

[55] Harpham T., Reichenheim M., Oser R., Thomas E., Hamid N., Jaswal S., Ludermir A., Aidoo M. (2003). Measuring mental health in a cost-effective manner. Health Policy Plan..

[56] Foa E.B., Riggs D.S., Dancu C.V., Rothbaum B.O. (1993). Reliability and validity of a brief instrument for assessing post-traumatic stress disorder. J. Trauma Stress.

[57] Stein D.J., Koen N., Donald K., Adnams C.M., Koopowitz S., Lund C., Marais A., Myers B., Roos A., Sorsdahl K. (2015). Investigating the psychosocial determinants of child health in africa: The drakenstein child health study. J. Neurosci. Methods.

[58] Budree S., Stein D., Brittain K., Goddard E., Koen N., Barnett W., Myer L., Zar H. (2017). Maternal and infant factors had a significant impact on birthweight and longitudinal growth in a south african birth cohort. Acta Paediatr..

[59] Draper N.R., Smith H. (2014). Applied Regression Analysis.

[60] Faust K., Raes J. (2012). Microbial interactions: From networks to models. Nat. Rev. Microbiol..

[61] Jiménez E., Delgado S., Fernández L., García N., Albújar M., Gómez A., Rodríguez J.M. (2008). Assessment of the bacterial diversity of human colostrum and screening of staphylococcal and enterococcal populations for potential virulence factors. Res. Microbiol..

[62] Ward T.L., Hosid S., Ioshikhes I., Altosaar I. (2013). Human milk metagenome: A functional capacity analysis. BMC Microbiol..

[63] Li S.-W., Watanabe K., Hsu C.-C., Chao S.-H., Yang Z.-H., Lin Y.-J., Chen C.-C., Cao Y.-M., Huang H.-C., Chang C.-H. (2017). Bacterial composition and diversity in breast milk samples from mothers living in taiwan and mainland china. Front. Microbiol..

[64] Hermansson H., Kumar H., Collado M.C., Salminen S., Isolauri E., Rautava S. (2019). Breast milk microbiota is shaped by mode of delivery and intrapartum antibiotic exposure. Front. Nutr..

[65] Deschasaux M., Bouter K.E., Prodan A., Levin E., Groen A.K., Herrema H., Tremaroli V., Bakker G.J., Attaye I., Pinto-Sietsma S.-J. (2018). Depicting the composition of gut microbiota in a population with varied ethnic origins but shared geography. Nat. Med..

[66] Sakwinska O., Moine D., Delley M., Combremont S., Rezzonico E., Descombes P., Vinyes-Pares G., Zhang Y., Wang P., Thakkar S.K. (2016). Microbiota in breast milk of chinese lactating mothers. PLoS ONE.

[67] Chen P.W., Lin Y.L., Huang M.S. (2018). Profiles of commensal and opportunistic bacteria in human milk from healthy donors in Taiwan. J. Food Drug Anal..

[68] Fitzstevens J.L., Smith K.C., Hagadorn J.I., Caimano M.J., Matson A.P., Brownell E.A. (2017). Systematic review of the human milk microbiota. Nutr. Clin. Pract..

[69] Collado M.C., Delgado S., Maldonado A., Rodríguez J.M. (2009). Assessment of the bacterial diversity of breast milk of healthy women by quantitative real-time pcr. Lett. Appl. Microbiol..

[70] Khodayar-Pardo P., Mira-Pascual L., Collado M.C., Martínez-Costa C. (2014). Impact of lactation stage, gestational age and mode of delivery on breast milk microbiota. J. Perinatol..

[71] Fernández L., Langa S., Martín V., Jiménez E., Martín R., Rodríguez J.M. (2013). The microbiota of human milk in healthy women. Cell. Mol. Biol..

[72] Fernandez M.F., Boris S., Barbes C. (2003). Probiotic properties of human lactobacilli strains to be used in the gastrointestinal tract. J. Appl. Microbiol..

[73] Fan W., Huo G., Li X., Yang L., Duan C. (2014). Impact of diet in shaping gut microbiota revealed by a comparative study in infants during the first six months of life. J. Microbiol. Biotechnol..

[74] Ma Z., Guan Q., Ye C., Zhang C., Foster J.A., Forney L.J. (2015). Network analysis suggests a potentially ‘evil’ alliance of opportunistic pathogens inhibited by a cooperative network in human milk bacterial communities. Sci. Rep..

[75] Mediano P., Fernández L., Jiménez E., Arroyo R., Espinosa-Martos I., Rodríguez J.M., Marín M. (2017). Microbial diversity in milk of women with mastitis: Potential role of coagulase-negative staphylococci, viridans group streptococci, and corynebacteria. J. Hum. Lact..

[76] Cacho N.T., Harrison N.A., Parker L.A., Padgett K.A., Lemas D.J., Marcial G.E., Li N., Carr L.E., Neu J., Lorca G.L. (2017). Personalization of the microbiota of donor human milk with mother’s own milk. Front. Microbiol..

[77] Li C., Gonzalez E., Solomons N., Scott M.E., Koski K. (2017). Human breast milk microbiota is influenced by maternal age and bmi, stage of lactation and infant feeding practices. FASEB J..

[78] Naseribafrouei A., Hestad K., Avershina E., Sekelja M., Linløkken A., Wilson R., Rudi K. (2014). Correlation between the human fecal microbiota and depression. Neurogastroenterol. Motil..

[79] Bendtsen K.M.B., Krych L., Sørensen D.B., Pang W., Nielsen D.S., Josefsen K., Hansen L.H., Sørensen S.J., Hansen A.K. (2012). Gut microbiota composition is correlated to grid floor induced stress and behavior in the balb/c mouse. PLoS ONE.

[80] Shen Y., Xu J., Li Z., Huang Y., Yuan Y., Wang J., Zhang M., Hu S., Liang Y. (2018). Analysis of gut microbiota diversity and auxiliary diagnosis as a biomarker in patients with schizophrenia: A cross-sectional study. Schizophr. Res..

[81] He Y., Wu W., Zheng H.-M., Li P., McDonald D., Sheng H.-F., Chen M.-X., Chen Z.-H., Ji G.-Y., Zheng Z.-D.-X. (2018). Regional variation limits applications of healthy gut microbiome reference ranges and disease models. Nat. Med..

[82] Gaulke C.A., Sharpton T.J. (2018). The influence of ethnicity and geography on human gut microbiome composition. Nat. Med..

