# Supplementary material for: Influence of Socio-Economic and Psychosocial Profiles on the Human Breast Milk Bacteriome of South African Women
Source: Nutrients. 2019 Jun 20;11(6):1390. doi: 10.3390/nu11061390 (PMC6627120; doi:10.3390/nu11061390)
Supplement: Supplementary file 1 [file nutrients-11-01390-s001.zip › Figure S3.pdf]

A

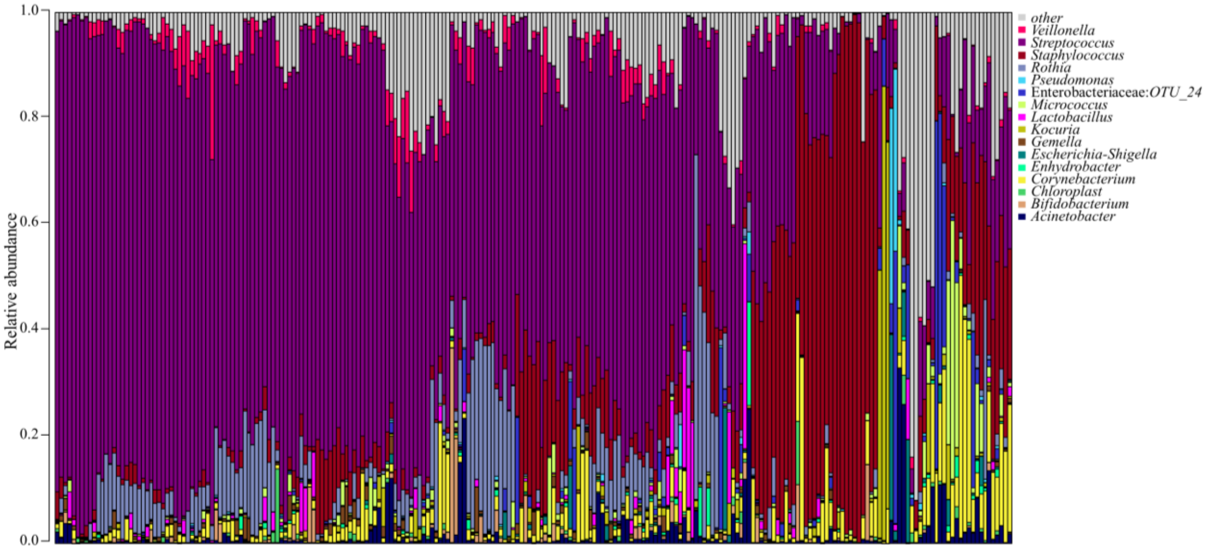

B

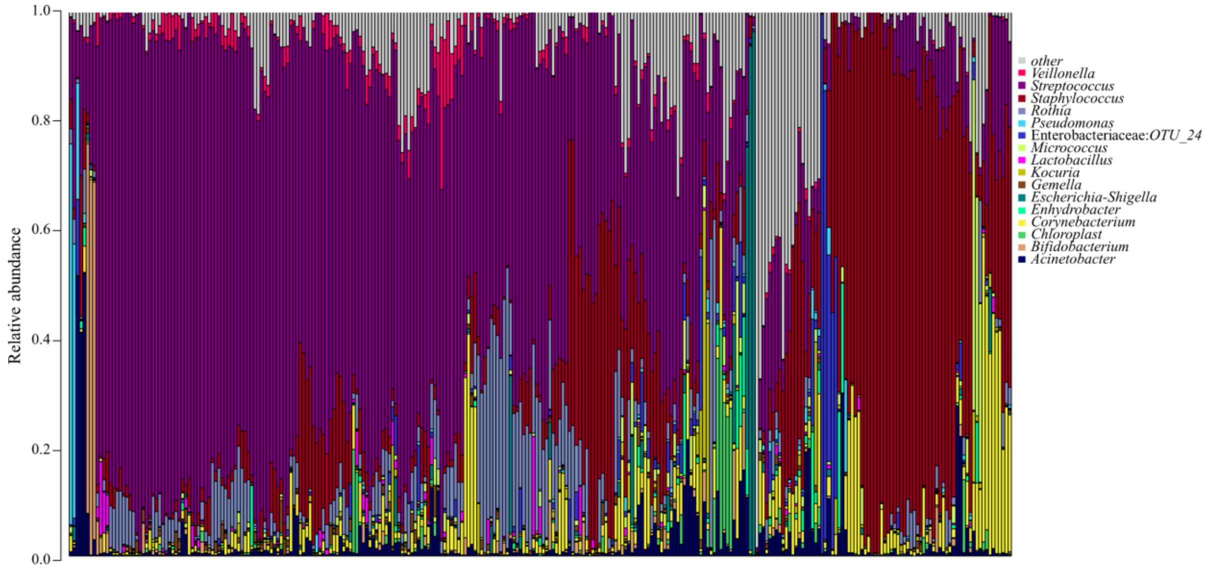

Figure S3. Bar-plot of relative abundances of bacteria genera. (**A)** Complete linkage unsupervised hierarchical clustering of bacterial abundances within the human breast milk bacteriome at Mbekweni study site (n =235). **(B)** Complete linkage unsupervised hierarchical clustering of bacterial abundances within the human breast milk bacteriome at TC Newman study site (n =287). Each bar represents a mother's HBM bacteriome profile and each coloured box, a bacterial genus. Genera with less than 0.5% abundance in a given sample are grouped together, herein referred to as "other" (grey boxes).
