# Supplementary material for: Influence of Socio-Economic and Psychosocial Profiles on the Human Breast Milk Bacteriome of South African Women
Source: Nutrients. 2019 Jun 20;11(6):1390. doi: 10.3390/nu11061390 (PMC6627120; doi:10.3390/nu11061390)
Supplement: Supplementary file 1 [file nutrients-11-01390-s001.zip › Figure S4.pdf]

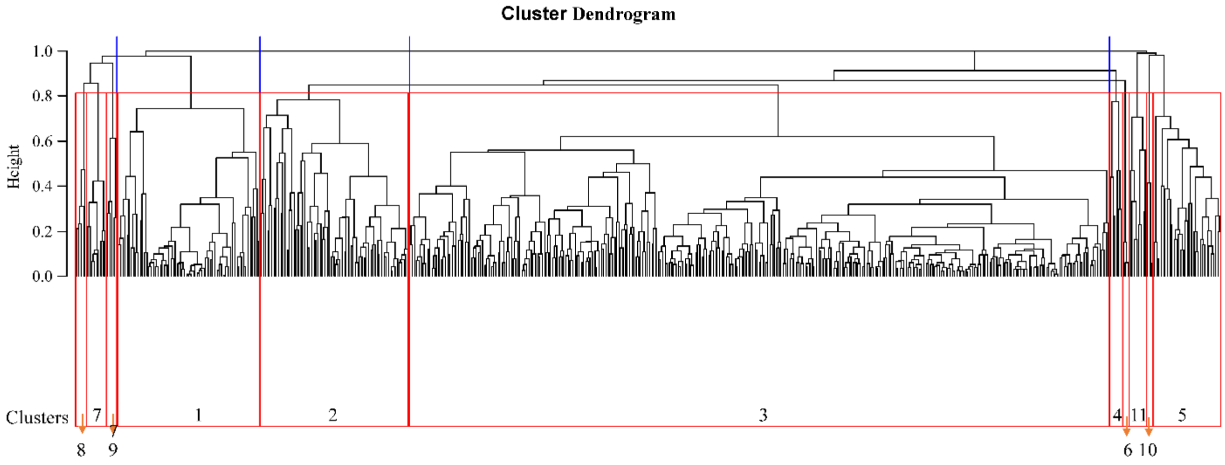

Figure S4. Dendogram of relative abundances of bacteria in HBM samples at genus level. Hierarchical complete linkage unsupervised clustering on Bray-Curtis dissimilarities shows 11 clusters at a clustering of height 0.8. Cluster 1 (65 members), cluster 2(68 members), cluster 3 (319 members), cluster 4 (6 members), cluster 5 (31 members), cluster 6 (3 members), cluster 7 (9 members), cluster 8 (5 members), cluster 9 (5 members), cluster 10 (3 members), and cluster 11 (8 members).
