# Supplementary material for: Influence of Socio-Economic and Psychosocial Profiles on the Human Breast Milk Bacteriome of South African Women
Source: Nutrients. 2019 Jun 20;11(6):1390. doi: 10.3390/nu11061390 (PMC6627120; doi:10.3390/nu11061390)
Supplement: Supplementary file 1 [file nutrients-11-01390-s001.zip › Figure S5.pdf]

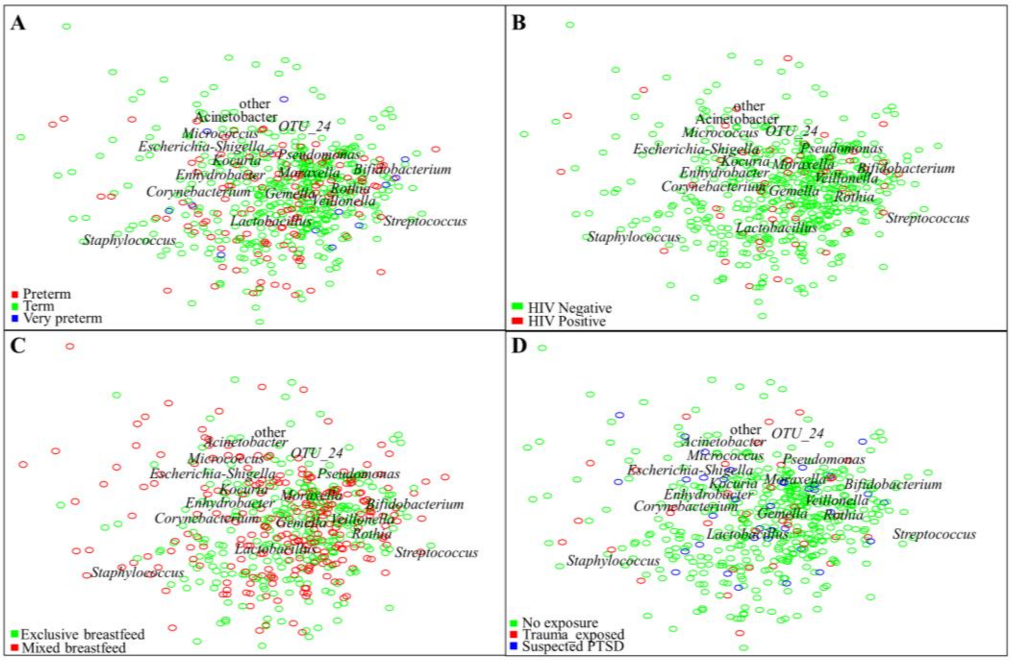

Figure S5. Log ratio biplot of human breast milk bacterial abundances at the genus level. Samples are colored according to **(A)** gestational age, **(B)** maternal smoking, **(C)** Body Mass Index (BMI), and **(D)** delivery mode. Each sample is represented by a circle and is colored based on group type. Samples (circles) that cluster together are similar in bacteria composition and abundance.
