# Supplementary material for: Influence of Socio-Economic and Psychosocial Profiles on the Human Breast Milk Bacteriome of South African Women
Source: Nutrients. 2019 Jun 20;11(6):1390. doi: 10.3390/nu11061390 (PMC6627120; doi:10.3390/nu11061390)
Supplement: Supplementary file 1 [file nutrients-11-01390-s001.zip › Figure S6.pdf]

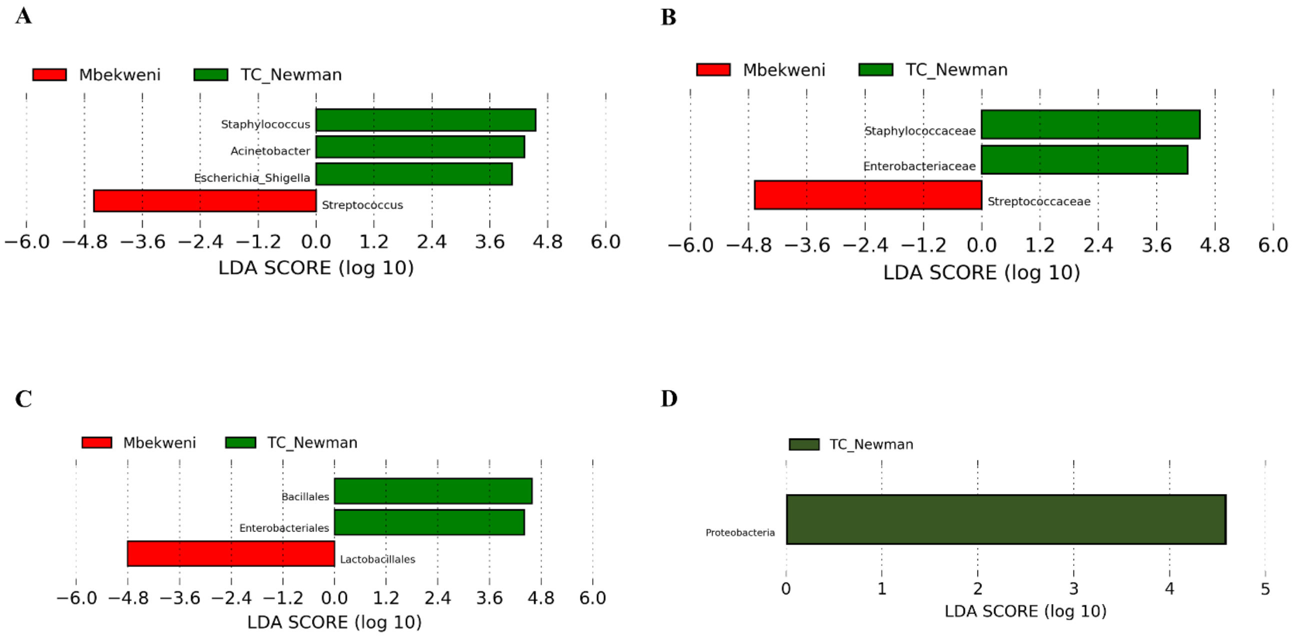

Figure S6. LEFSe plots showing differences between the HBM bacteriome of participants based on study site. The histograms show the linear discriminant analysis (LDA) scores of bacteriome at **(A)** genus, **(B)** family, **(C)** order and **(D)** phylum level. The higher the LDA score, the more likely it is that the specific taxon is differentially abundant in one group compared to the other group. HBM samples of mothers from Mbekweni study site have higher relative abundance of the taxa shown in red, while HBM samples of mothers from TC Newman study site have higher relative abundance of the taxa shown in green. LEFSe=Linear Discriminant Analysis Effect Size.
