# Supplementary material for: Influence of Socio-Economic and Psychosocial Profiles on the Human Breast Milk Bacteriome of South African Women
Source: Nutrients. 2019 Jun 20;11(6):1390. doi: 10.3390/nu11061390 (PMC6627120; doi:10.3390/nu11061390)
Supplement: Supplementary file 1 [file nutrients-11-01390-s001.zip › Figure S7.pdf]

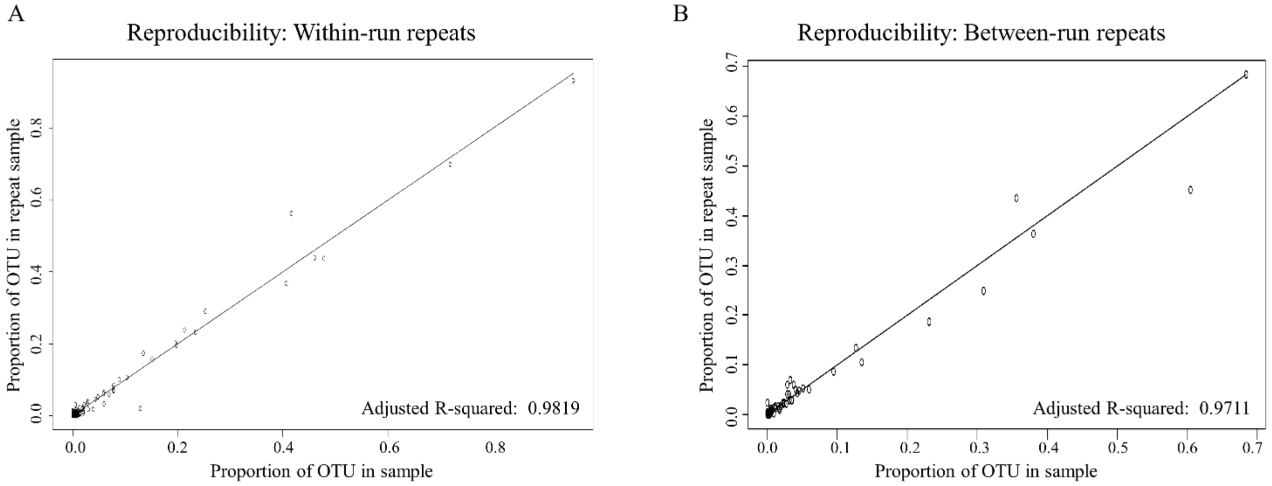

Figure S7. 16S rRNA gene sequencing reproducibility. Scatter plots represent the relative abundance of each OTU from **(A)** seven within-run repeats and **(B)** four between-run repeats together with their technical repeats. A multiple R-square which is the proportion of variance reproduced by replicating the reads is generated.
