# Supplementary material for: Influence of Socio-Economic and Psychosocial Profiles on the Human Breast Milk Bacteriome of South African Women
Source: Nutrients. 2019 Jun 20;11(6):1390. doi: 10.3390/nu11061390 (PMC6627120; doi:10.3390/nu11061390)
Supplement: Supplementary file 1 [file nutrients-11-01390-s001.zip › Figure S8.pdf]

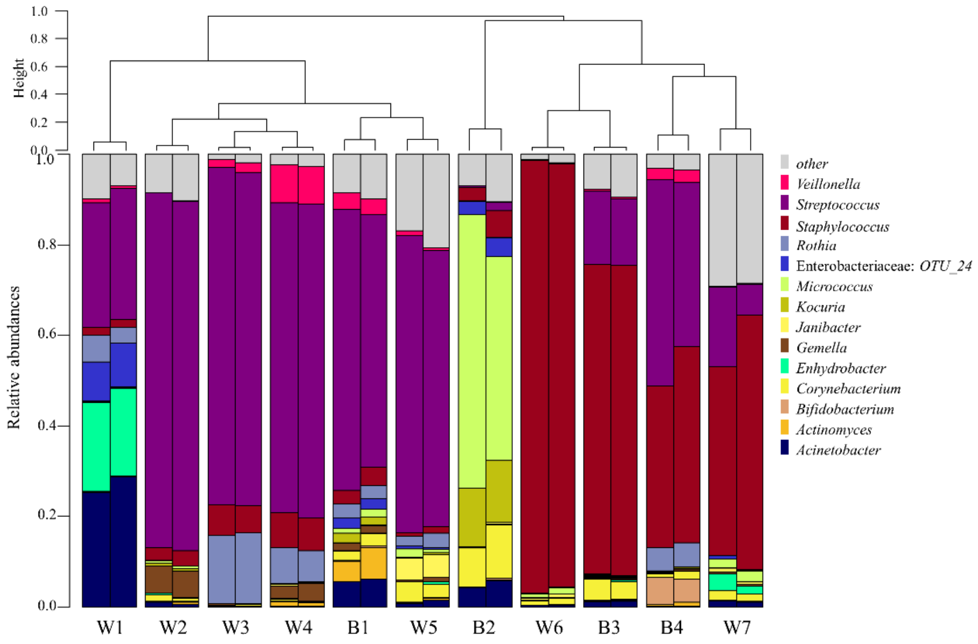

Figure S8. Bar-plot of relative abundances of bacteria genera in human breast milk (HBM) samples and their technical repeats. Complete linkage unsupervised hierarchical clustering of human breast milk bacterial profiles within a sample and its repeat (paired bar-plots). Each bar represents a sample and each coloured box, a bacteria genus. Genera with less than 0.5% abundance in a given sample are grouped together, herein referred to as “other” (grey boxes). W-Within run repeat, B-Between run repeat.
