# Supplementary figures and images for: Influence of Socio-Economic and Psychosocial Profiles on the Human Breast Milk Bacteriome of South African Women
Source: Nutrients. 2019 Jun 20;11(6):1390. doi: 10.3390/nu11061390 (PMC6627120; doi:10.3390/nu11061390)

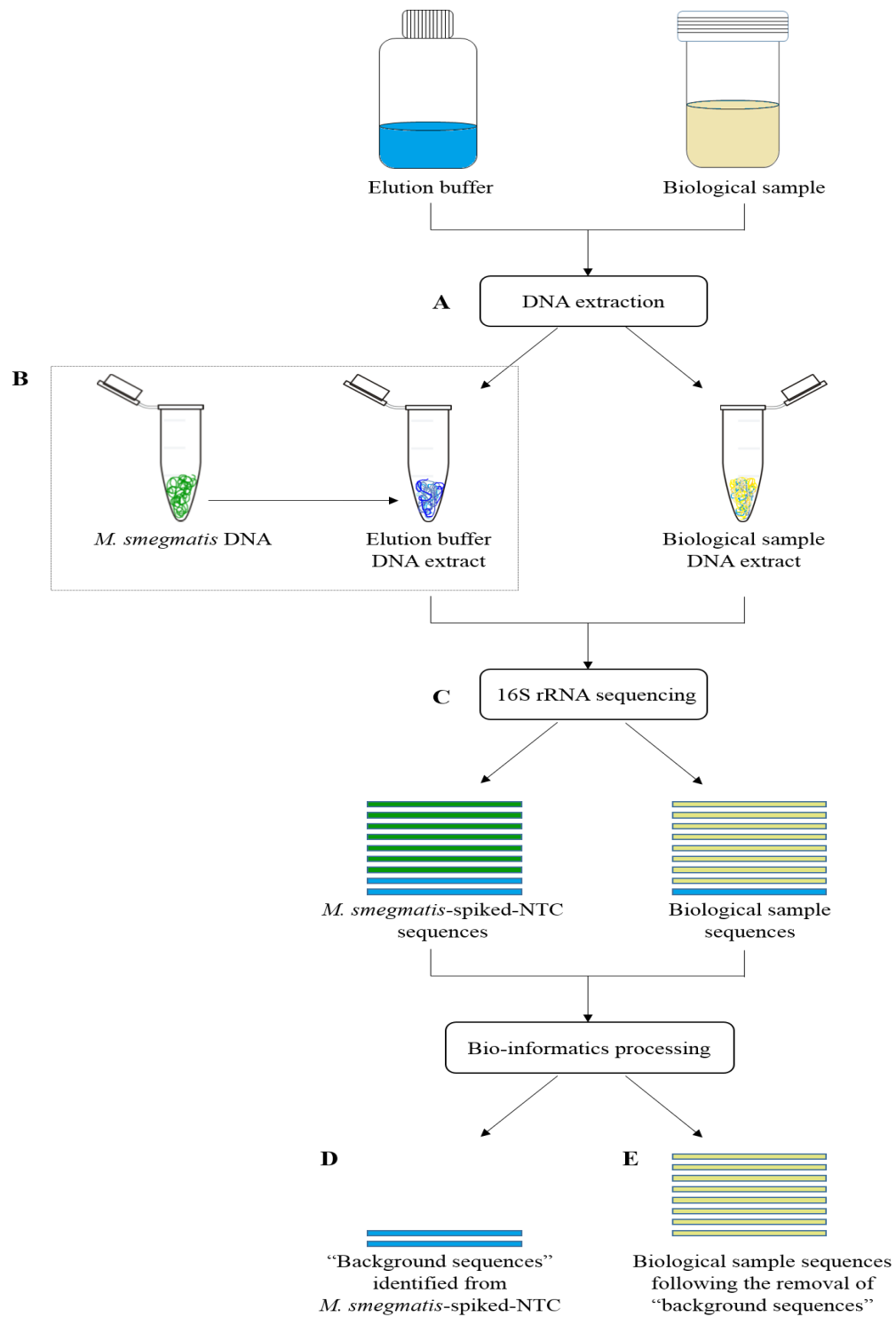

**Figure S1.** In-silico de-contamination of biological human breast milk samples.

Supplement: Supplementary file 1 [file nutrients-11-01390-s001.zip › Figure S1.pdf]
